# Photodynamic Antibacterial Therapy of Gallic Acid-Derived Carbon-Based Nanoparticles (GACNPs): Synthesis, Characterization, and Hydrogel Formulation

**DOI:** 10.3390/pharmaceutics16020254

**Published:** 2024-02-09

**Authors:** Koranat Dechsri, Cheewita Suwanchawalit, Prasopchai Patrojanasophon, Praneet Opanasopit, Supusson Pengnam, Thapakorn Charoenying, Theerada Taesotikul

**Affiliations:** 1 Pharmaceutical Development of Green Innovations Group (PDGIG), Faculty of Pharmacy, Silpakorn University, Nakhon Pathom 73000, Thailand; dechsri_k@su.ac.th (K.D.); patrojanasophon_p@su.ac.th (P.P.); opanasopit_p@su.ac.th (P.O.); pengnam_s@su.ac.th (S.P.); 2 Department of Chemistry, Faculty of Science, Silpakorn University, Nakhon Pathom 73000, Thailand; suwanchawalit_c@su.ac.th; 3 Department of Biomedicine and Health Informatics, Faculty of Pharmacy, Silpakorn University, Nakhon Pathom 73000, Thailand

**Keywords:** antibacterial activity, carbon-based nanoparticles, gallic acid, hydrogel, photodynamic therapy

## Abstract

Carbon-based nanoparticles (CNPs) have gained recognition because of their good biocompatibility, easy preparation, and excellent phototherapy properties. In biomedicine applications, CNPs are widely applied as photodynamic agents for antibacterial purposes. Photodynamic therapy has been considered a candidate for antibacterial agents because of its noninvasiveness and minimal side effects, especially in the improvement in antibacterial activity against multidrug-resistant bacteria, compared with conventional antibiotic medicines. Here, we developed CNPs from an active polyhydroxy phenolic compound, namely, gallic acid, which has abundant hydroxyl groups that can yield photodynamic effects. Gallic acid CNPs (GACNPs) were rapidly fabricated via a microwave-assisted technique at 200 °C for 20 min. GACNPs revealed notable antibacterial properties against Gram-positive and Gram-negative bacteria, including *Staphylococcus aureus* (*S. aureus*) and *Escherichia coli* (*E. coli*). The minimum inhibitory concentrations of GACNPs in *S. aureus* and *E. coli* were equal at approximately 0.29 mg/mL and considerably lower than those in gallic acid solution. Furthermore, the GACNP-loaded hydrogel patches demonstrated an attractive photodynamic effect against *S. aureus*, and it was superior to that of Ag hydrofiber^®^, a commercial material. Therefore, the photodynamic properties of GACNPs can be potentially used in the development of antibacterial hydrogels for wound healing applications.

## 1. Introduction

Infectious diseases caused by multidrug-resistant bacteria are one of the most widespread problems worldwide; they cause massive challenges in the development of effective antibiotic drugs to replace conventional antibacterial [1,2,3,4,5]. In the past decade, nanoparticles (NPs) have been investigated to improve their antibacterial activity [6,7,8]. NPs are applied in various biomedical applications, such as biosensing, bioimaging, drug delivery systems, and therapeutics (antiviral, antifungal, anticancer, and antibacterial agents) [9]. Abundant research has focused on the development of NPs, such as silver NPs, gold NPs, titanium dioxide, magnetite NPs, zinc oxide NPs, and carbon-based NPs (CNPs), especially in antibacterial applications [10,11,12]. CNPs are recognized because of their unique characteristics, including nanoscale range, excellent biocompatibility, low toxicity, low cost, water dispersibility, environmental friendliness, and easy synthesis and functionalization [13,14,15,16].

CNPs can be prepared through various methods which are mainly divided into two technologies: (1) top-down (laser ablation, ultrasound, and electrochemistry) and (2) bottom-up (microwave digestion, thermal decomposition, ultrasound, and hydrothermal method) technologies [17,18,19,20]. However, top-down technology has limitations, such as expensive and complicated equipment and higher toxicity of the attained NPs than bottom-up technology, which is more convenient and has a shorter reaction time [21]. In addition, bottom-up technology can be used to control the particle size at a certain distribution, power, and temperature throughout the synthesis, leading to rapid utilization. Therefore, bottom-up technology is more attractive and more frequently used than other technologies [22,23,24]. Furthermore, suitable precursors, including bulk carbon materials, natural polymers, synthetic polymers, biomass, and small molecules, are used as carbon sources [25].

Gallic acid is one of the most compelling small molecules with an active polyhydroxy phenolic compound, and it has been applied in various biological applications, such as antidiabetic, antioxidant, anti-inflammatory, anticancer, gastroprotective, and antibacterial agents [26,27]. Various researchers have proposed taking advantage of the antibacterial activity of gallic acid. The antibacterial mechanisms of gallic acid include bacterial-membrane pore formation, increased membrane permeability, and changes in the physicochemical properties of bacteria [28,29]. In addition, gallic acid can penetrate bacterial cell membranes, resulting in the inhibition of cell wall synthesis and biofilm formation, protein and enzyme inactivation, DNA damage, and oxidative stress [30,31]. Hence, gallic acid was selected as a precursor for the synthesis of CNPs. Gallic acid was altered because CNPs can increase antibacterial activity, which is consistent with the findings of previous studies [32]. Moreover, because of their low toxicity, the previous research utilized gallic acid-loaded graphene oxide against methicillin-resistant *Staphylococcus aureus* (MRSA) strains, which showed low toxicity of nanocomposites at more than 500 μg/mL for up to 72 h in zebrafish embryos [33].

Antibacterial photodynamic therapy (PDT) has increased the antibacterial activity of CNPs [34,35,36]. CNPs can act as photosensitizers which possess excellent activity for fighting bacterial resistance. Photoexcited CNPs can induce the formation of reactive oxygen species (ROS) through the photochemical and photophysical mechanisms explained by the Jablonski diagram [37,38,39]. The activity of photoexcited CNPs occurs when they absorb light with a suitable wavelength and generate ROS, such as hydroxyl radical (OH•) and singlet molecular oxygen (^1^O_2_) [40,41,42,43]. ROS activation can damage intracellular biomolecules, which affects the expression of genes and finally leads to bacterial death [44]. Hence, the phototherapeutic properties of CNPs are attractive for potential antibacterial applications.

Studies have been increasingly focusing on pharmaceutical dosage forms to improve and expand various pharmaceutical products with the aim of enhancing their efficiency, safety, and convenience of use [45]. Hydrogel wound dressings are wound-healing pharmaceutical products that are flexible and suitable for wound areas. Furthermore, hydrogel patches retain the moisture of wounds better than other products [46]. Hydrogel patches are three-dimensional (3D) cross-linked polymer structures. These materials are attractive and applicable in advertising because of their ideal characteristics, including a moist environment around the wound, exudate absorption, nontoxicity, biocompatibility, and easy preparation [47,48]. Freeze–thaw is a physical cross-linking technique utilized in hydrogel fabrication characterized by two distinct stages: freezing of the precursor solution at −20 °C, followed by thawing at room temperature. This method aims to precisely regulate the ice crystallization process during freezing and the subsequent formation of an ordered structure during thawing, thereby imparting optimal properties on the resulting hydrogels. Hydrogels consist of polymers carefully chosen for their exceptional properties and suitability for various applications [49]. Polyvinyl alcohol (PVA) is a commonly used polymer for hydrogel fabrication because of its distinguished properties and excellent biocompatibility. In addition, PVA has been diversely applied to biomedical applications, such as hemostasis bandages, self-healing materials, 3D tissue scaffolds, and wound healing [50,51,52,53].

In this research, gallic acid was selected as a precursor and synthesized as gallic acid CNPs (GACNPs) through microwave pyrolysis with a short reaction time [21]. Nowadays, there are various studies on synthesized carbon-based nanoparticles for biomedical fields. They usually utilize hydrothermal treatment for carbon-based nanoparticle synthesis. This method is time- and energy-consuming, normally in the range of 2–13 h [51,54,55]. Our research utilized microwave pyrolysis, which usually requires a short reaction time of 20 min. Consequently, our research has the advantage of saving time in the synthesis process. The NPs were collected to investigate their morphology, particle size, polydispersity index (PDI), and zeta potential. In addition, attenuated total reflectance Fourier transform infrared (ATR-FTIR) and X-ray diffraction (XRD) were used to confirm the structure of GACNPs. After determining the optimal conditions for GACNP synthesis, their PDTs and antibacterial activity, including minimum inhibitory concentration (MIC) and minimum bactericidal concentration (MBC), were investigated. Finally, GACNPs were loaded into suitable hydrogels, and their characteristics, including the chemical and mechanical properties, water content, and water absorption photodynamic antibacterial therapy, were further evaluated and compared with those of blank hydrogels.

## 2. Materials and Methods

### 2.1. Materials

Chemical reagents including gallic acid monohydrate (assay > 98%, by HPLC) were obtained from Fluka Chemie AG (Buchs, Switzerland), and PVA (molecular weight 60 kDa, degree of hydrolysis = 98.1%) was obtained from Sigma-Aldrich (St. Louis, MO, USA). Tryptic soy agar (TSA) and tryptic soy broth (TSB), which were used as growth media for microorganisms, were purchased from Merck KGaA (Darmstadt, Germany) and Becton, Dickinson, and Company (Franklin Lakes, NJ, USA), respectively. The Gram-positive *Staphylococcus aureus* ATCC 6538P (*S. aureus*) and Gram-negative *Escherichia coli* DMST 4212 (*E. coli*) were used to test the antibacterial activity. Ultrapure water was prepared using a Milli-Q system (Millipore, Burlington, MD, USA).

### 2.2. Synthesis of GACNPs

**Figure 1:**
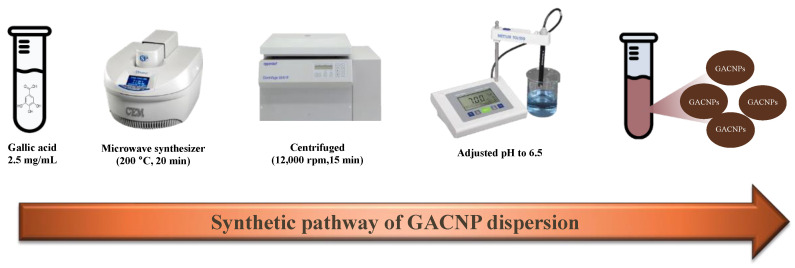
Synthetic pathway of GACNP dispersion.

GACNPs were synthesized through microwave pyrolysis with gallic acid as a carbon source. The synthesis scheme is presented in Figure 1. Gallic acid was dissolved in deionized water to obtain a final concentration of 2.5 mg/mL. Next, 20 mL solution was transferred to a reaction vessel, which was then transferred to a microwave for GACNP synthesis (Discover SP, CEM Corporation, Matthews, NC, USA). The optimal reaction conditions for the preparation of GACNPs, including the reaction temperature and time, were investigated. The as-prepared GACNPs were centrifuged at 12,000 rpm (19,802 rcf) for 15 min to remove large NPs. Afterward, the pH of the GACNP dispersion was adjusted to 6.5. The GACNP dispersion was kept in a refrigerator at 4 °C for future use.

### 2.3. Characterization of GACNPs

The particle size, PDI, and zeta potential of GACNPs were measured using a dynamic light scattering (DLS) analyzer (Zetasizer Nano-ZS, Malvern Instruments, Malvern, UK). A scanning electron microscope (SEM; MIRA 3, Tescan, Brno, Czech Republic) was used to investigate the morphology of GACNPs.

ATR-FTIR spectra were collected using a Nicolet iS5 FTIR spectrometer (Thermo Fisher Scientific, Waltham, MA, USA). All spectra were captured in 16 running scans and at 4 cm^−1^ resolutions to acquire spectral results at wavenumbers ranging from 500 cm^−1^ to 4000 cm^−1^.

The XRD pattern of GACNPs was measured on an X-ray diffractometer (Malvern PANalytical, Aeris, Malvern, UK) at a scanning rate of 2°/min in the range of 5–60.

The thermogravimetric analysis (TGA) of GACNPs was investigated using a Simultaneous Thermal Analyzer (STA) 6000 (Perkin-Elmer, MA, USA) by heating to 600 °C at a heating rate of 10 °C/min in an atmosphere of nitrogen gas.

### 2.4. Antibacterial Activity of GACNPs

#### 2.4.1. Preparation of Bacterial Suspensions

The antibacterial activity against *S. aureus* and *E. coli* was assessed using broth microdilution assay in a 24-well plate. The bacterial suspensions were prepared in TSB and incubated at 37 °C for 24 h. Then, the turbidity of bacterial suspensions was adjusted to attain a value equivalent to a 0.5 McFarland standard, which was used as a reference in the measurement of optical density (OD) at 600 nm.

#### 2.4.2. Minimum Inhibitory Concentration

A twofold serial dilution of the GACNP dispersion with TSB medium was prepared to obtain various concentrations of the samples. Subsequently, 10 µL of bacterial suspension was added to 990 µL of the prepared suspensions to obtain the final bacterial concentration (10^6^ CFU/mL). The bacterial suspensions and blank TSB medium were used as controls. Thereafter, the mixtures were incubated at 37 °C for 24 h to determine the MIC of GACNPs in the clear solution with the lowest GACNP concentration.

#### 2.4.3. Minimum Bactericidal Concentration

A certain amount of clear GACNP dispersion from the MIC study was selected for MBC investigation using the spread plate method. In brief, 100 µL samples were spread onto TSA plates, which were then incubated at 37 °C for 24 h. The untreated bacteria were used as controls. Clear agar plates without any colonies were observed for MBC.

#### 2.4.4. Photodynamic Antibacterial Activity of GACNPs

The photodynamic antibacterial activity of GACNPs was investigated using the spread plate method that involved counting the colonies. *S. aureus* and *E. coli* which were used as test microorganisms. Briefly, bacterial suspensions were added to the mixture of TSB medium and GACNP dispersion to obtain the final bacterial concentration (10^6^ CFU/mL) in a 500 µL reaction. Then, 100 µL mixtures were spread onto the TSA plates. Sterile water was used as the control group. All TSA plates were then incubated at 37 °C for 3 h. The plates were then irradiated with a light-emitting diode (LED) lamp at 350–700 nm (visible light, 0.12 W/cm^2^) for 10 min. Next, the plates were incubated for 18 h. Finally, the results of irradiation and non-irradiation were compared through observation of the colony number.

#### 2.4.5. Detection of the Formation of Singlet Oxygen (^1^O_2_) of GACNPs

1,3-diphenylisobenzofuran (DPBF) was applied as a trapping agent to detect a singlet oxygen (^1^O_2_). The method was modified from the previous study [56]. Briefly, DPBF was dissolved in ethanol solution to obtain the final concentration at 1.35 mg/mL. A total of 50 μL of DPBF solution was then mixed with 200 μL of GACNP dispersion (300 μg/mL). After that, the mixture was exposed to visible light (0.12 W/cm^2^) for 10 min. Then, 100 μL of the mixture solution was put into a 96-well plate to measure the absorbance of DPBF at 430 nm with a microplate reader.

### 2.5. Preparation of Hydrogels

#### 2.5.1. Preparation of Blank Hydrogels

Hydrogels were fabricated using 15% *w*/*w* PVA via a freeze–thaw technique. Briefly, PVA powder was dissolved in deionized water at 90 °C with slow stirring for 4 h to obtain a clear gel. The gel was poured into molds of various thicknesses (1.7, 2.2, and 2.7 mm) and allowed to stand at ambient temperature for 1 h. The freeze–thaw process was performed for four cycles. Each cycle consisted of freezing and thawing at −20 °C for 18 h and at ambient temperature for 6 h. An optimal blank hydrogel was selected for the loading of GACNPs.

#### 2.5.2. Preparation of GACNP-Loaded Hydrogels

**Figure 2:**
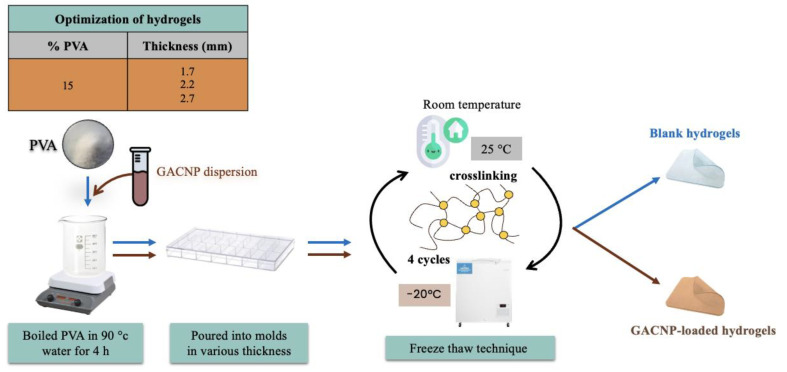
Schematic illustration of the preparation of blank hydrogels and GACNP-loaded hydrogels.

GACNP-loaded hydrogels were prepared through the addition of GACNP dispersion, instead of the deionized water used during the dissolution of PVA powder by GACNPs of various weights, to the hydrogel patches at 0.5, 0.7, and 0.9 mg/g. The other process was performed in accordance with a previous method. The schematic illustration of the preparation of blank hydrogels and GACNP-loaded hydrogels is presented in Figure 2.

### 2.6. Characterization of Hydrogels

#### 2.6.1. Chemical Properties

The components of hydrogel patches were recorded using ATR-FTIR equipment (Nicolet iS5, Thermo Fisher Scientific, Waltham, MA, USA). The hydrogel patches were cut into squares with a width and length of 1 cm. The decorated hydrogel patches were then heated at 60 °C for 4 h. Then, the FTIR spectra of GACNP-loaded hydrogel patches were examined from 500 cm^−1^ to 4000 cm^−1^ in 16 running scans and at 4 cm^−1^ resolutions.

#### 2.6.2. Mechanical Properties

The tensile strength of blank hydrogels was examined using a texture analyzer (TA.XT plus, Stable Micro Systems, Surrey, UK) with a 5 kg load cell. Furthermore, Young’s modulus and elongation were measured using tensile grips at a constant test speed of 5.0 mm/s until the blank hydrogel patches were torn. The blank hydrogel patches were cut into rectangles with a width of 1 cm and a length of 5 cm. The tensile strength, Young’s modulus, and elongation were computed using Equations (1)–(3), respectively:

(1) $$Tensile\;strength=\frac{Maximum\;force\;at\;breaking\;point}{Cross\;section\;area}$$

(2) $$Young’s\;modulus=\frac{Stress}{Strain}$$

(3) $$Elongation\left(\%\right)=\frac{Extension\;of\;lenght\;at\;breaking\;point}{Initial\;length}\times 100$$

#### 2.6.3. Water Content

The water contents of the blank hydrogel and GACNP-loaded hydrogel patches were investigated. The hydrogel patches were cut into squares with a width and a length of 1 cm. The decorated hydrogel patches were then weighed and heated at 60 °C in a hot air oven to attain a constant patch weight. The water contents were calculated using weight differences between the initial and constant weights of the hydrogel patches after heating. According to Equation (4), the water content can be computed as follows:

(4) $$Water\;content\left(\%\right)=\frac{W_{i}-W_{d}}{W_{i}}\times 100$$

where W_i_ and W_d_ refer to the weights of the initial and dried patches, respectively.

#### 2.6.4. Water Absorption

The water absorption of the blank hydrogel and GACNP-loaded patches was investigated. The hydrogel patches were cut into squares with a width and a length of 1 cm. The decorated hydrogel patches were then weighed, soaked in distilled water, and incubated at 37 °C for 24 h. Excess water from the swollen hydrogel surface was lightly removed using filter paper. The swollen hydrogel was calculated to determine the percentage of water absorption (Equation (5)):

(5) $$Water\;absorption\left(\%\right)=\frac{W_{s}-W_{i}}{W_{i}}\times 100$$

where W_i_ and W_s_ denote the weights of the initial and swollen patches, respectively.

#### 2.6.5. Stability Study

GACNP-loaded hydrogel patches were kept in moisture-proof aluminum foil bags at 5 °C, 25 °C, and 40 °C for 30 days. The characteristics of the GACNP-loaded hydrogel patches including mechanical properties (Young’s Modulus, tensile strength, and elongation), water content, and water absorption were measured according to the previous method.

### 2.7. Antibacterial Activity of GACNP-Loaded Hydrogel Patches

#### 2.7.1. Quantitative Method

Estimation of the relative antibacterial activity of GACNP-loaded hydrogel patches against *S. aureus* and *E. coli* was completed using a microplate reader (VICTOR Nivo^TM^ Multimode Plate Reader, PerkinElmer, Rodgau, Germany). Briefly, 10 µL of bacterial suspension was added to 990 µL TSB medium in a 24-well plate to obtain the final bacterial concentration of 10^6^ CFU/mL or a value equal to 0.5 McFarland standard. The decorated hydrogel patches (0.08 g/piece) were then added to the prepared suspensions in amounts of 1, 2, and 3 pieces per well, respectively. The plates were then incubated at 37 °C for 24 h. Subsequently, the medium absorbance at 600 nm was measured using a microplate reader. Bacterial suspensions without a hydrogel patch were used as a control group. The relative antibacterial activities of the samples compared with the control group were computed using Equation (6):

(6) $$Relative\;antibacterial\;activity\left(\%\right)=\frac{A_{C}-A_{S}}{A_{C}}\times 100$$

where A_c_ and A_s_ refer to the absorbances of the control and sample groups, respectively.

#### 2.7.2. Qualitative Method (Disc Diffusion Assay)

The antibacterial activity of GACNP-loaded hydrogel patches against *S. aureus* and *E. coli* was estimated using a disc diffusion assay. Briefly, bacterial suspensions with a density of 10^6^ CFU/mL or equal to 0.5 McFarland standard were prepared. Next, 50 µL of bacterial suspension was pipetted and gently spread over the agar plates. The GACNP-loaded hydrogel patches were modified into 0.6 cm^2^ circular patches with a weight of 0.08 g/piece. The hydrogel patches were then sterilized under ultraviolet light for 15 min per side. After complete sterilization, the decorated hydrogels were gently placed onto the agar plate, which was already spread with the bacterial suspension. Next, the plates were incubated at 37 °C for 3 h. After incubation, the plates were irradiated using an LED lamp at 350–700 nm (visible light, 0.124 W/cm^2^) for 10 min. Afterward, the plates were further incubated for 18 h. Finally, the diameter of the clear zone around the decorated hydrogels was measured to investigate the zone of inhibition (cm). The blank hydrogel patches and a commercial product (Ag hydrofiber^®^) were used as negative and positive controls, respectively.

### 2.8. Statistical Analysis

The results of each experiment were recorded in triplicate and reported as mean ± standard deviation. Independent *t*-test and F-test were conducted using IBM SPSS Statistics version 28 at a 95% confidence interval. Significant differences were defined at *p* < 0.05.

## 3. Results and Discussion

### 3.1. Synthesis of GACNPs

**Figure 3:**
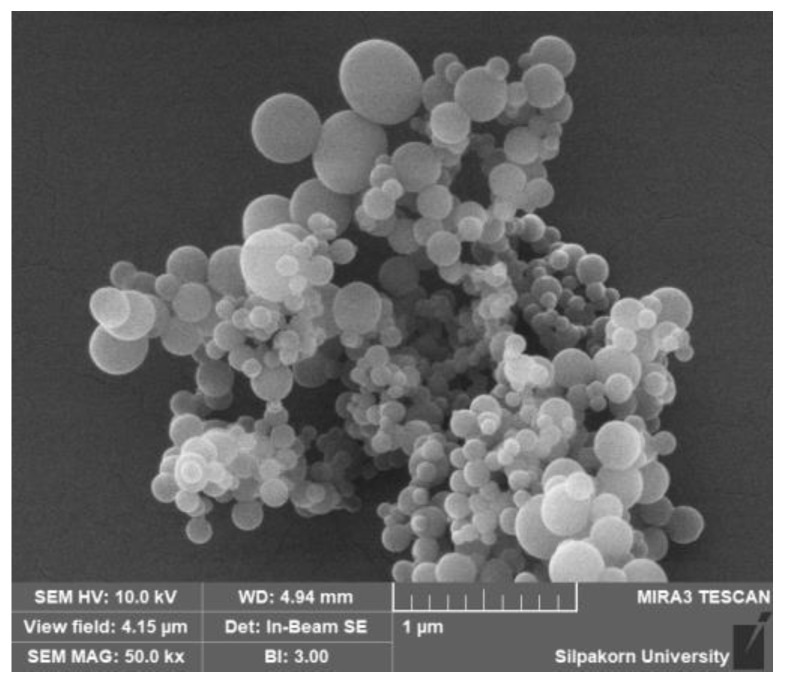
SEM images of GACNPs.

GACNPs were synthesized through microwave pyrolysis. The reaction time and temperature were screened to determine the optimal reaction conditions. The particle size, PDI, and zeta potential of the attained GACNPs were investigated through DLS measurements. The results are recorded in Table 1. For the effect of temperature, the optimal reaction time was fixed at 20 min, and the temperature was varied at 175 °C, 200 °C, and 220 °C. The results verify that the optimal reaction temperature was 200 °C. In the further investigation of the influence of temperature, when the temperature was lower or higher than 200 °C, a large particle formation was induced because the increase in reaction temperature decreased the particle size. However, when the reaction temperature was adequately high in excess, the NPs could agglomerate by generating more carbon core, leading to a large particle size [57]. To investigate the effect of time and temperature, when the time was increased to 10, 15, and 20 min at a fixed temperature of 200 °C, the particle size of GACNPs decreased continuously because the reaction could not complete synthesis before 20 min [58]. The results show that the smallest GACNPs were synthesized at 200 °C for 20 min. We can propose a mechanism for the formation of GACNPs: The initial reaction step is decarboxylation, which is catalyzed by heat, and a carboxyl group (–COOH) of gallic acid is removed to generate pyrogallol (PG), which could be a key intermediate for subsequent reactions leading to next step for the synthesis of GACNPs [59]. The second reaction step is the carbonization process. The final product rapidly self-polymerizes to form GACNPs through dehydration, cross-linking, and carbonization covalently linked with aggregation [60,61,62]. After the reaction is completed, the color of the gallic solution changed to the brown color of GACNP dispersion, which is shown in Figure S1. In addition, the morphology of GACNPs was investigated using a SEM (Figure 3). The figure reveals the spherical shape of GACNPs based on previous research [63].

### 3.2. Characterization of GACNPs

**Figure 4:**
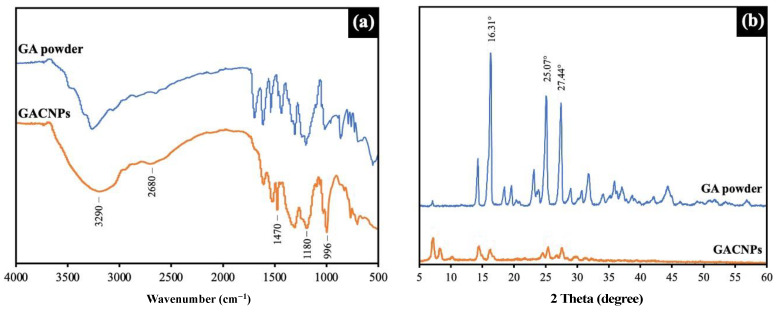
Characterization of GACNPs and GA powder: (**a**) ATR-FTIR and (**b**) XRD spectrum.

Figure 4a shows the ATR-FTIR spectra of GACNPs compared with those of gallic acid powder. The results reveal the distinguished chemical composition and functional group on the surface of GACNPs, which confirmed their formation after synthesis [64,65,66]. The broad band at 3290 cm^−1^ was assigned to the stretching vibration of –OH. The negative charge on the surface of GACNPs was confirmed through the measurement of zeta potential. The bond at 2680 cm^−1^ corresponds to C–H stretching. A strong bond at 1470 cm^−1^ demonstrates the presence of aromatic C=C, which can explain the aromatic skeletal hydrocarbon of GACNPs. The other peaks at 1180 and 996 cm^−1^ represent C–O–C stretching and C=C, respectively. Therefore, the ATR-FTIR spectra proved the structure of the as-prepared GACNPs [67].

Figure 4b displays the XRD pattern of GACNPs compared with that of gallic acid powder. The XRD patterns of gallic acid powder (blue line) exhibited outstanding sharp peaks at a 2θ value of 16.31°, 25.07°, and 27.44°, which corresponded to the crystalline structure of an organic small molecule [68]. The crystalline state of the GACNP structure was achieved after complete synthesis. The XRD pattern of GACNPs (orange line) revealed numerous intense peaks and signal-to-noise ratios of more than three peaks, which indicated the polycrystalline form of GACNPs. Furthermore, the existence of numerous narrow peaks demonstrated slight variations in the GACNP structure. The XRD pattern of GACNPs confirmed the evolution of CNPs with a crystalline nature [69,70].

The thermal degradation of GACNPs was investigated using the TGA technique, in which the degradation temperature and total weight loss are given. The result is presented in the Supplementary Materials file, as shown in Figure S3. According to the result, the GACNP decomposition was divided into two parts. The first decomposition process started at about 100–250 °C, which might be the elimination of water and the hydroxyl group. Next, the second decomposition step occurred at 250–600 °C, which might be the elimination of oxygen-bearing moieties including –CH. Moreover, the result showed that weight loss was not completed below 600 °C, which indicates that the GACNPs presented high thermal stability as well as stable interactions within the GACNP conjugation [71,72,73].

### 3.3. Photodynamic Antibacterial Activity of GACNPs

The antibacterial activity was investigated through broth microdilution assay and the spread plate method to obtain the MIC and MBC, respectively. As shown in Table 2, the concentrations (MIC and MBC) against *S. aureus* and *E. coli* were considerably lower for the GACNPs compared to the gallic acid solution. The antibacterial mechanisms of gallic acid include bacterial cell membrane penetration, which results in the inhibition of cell wall synthesis, protein and enzyme inactivation, DNA damage, and oxidative stress induction. GACNP synthesis can synergize the antibacterial activity with the precursor (gallic acid) because the nano-range particle size of GACNPs may induce distribution through the lipid bilayer of the bacterial cell membrane more easily than the gallic acid solution and present more attractive antibacterial activity [74].

**Figure 5:**
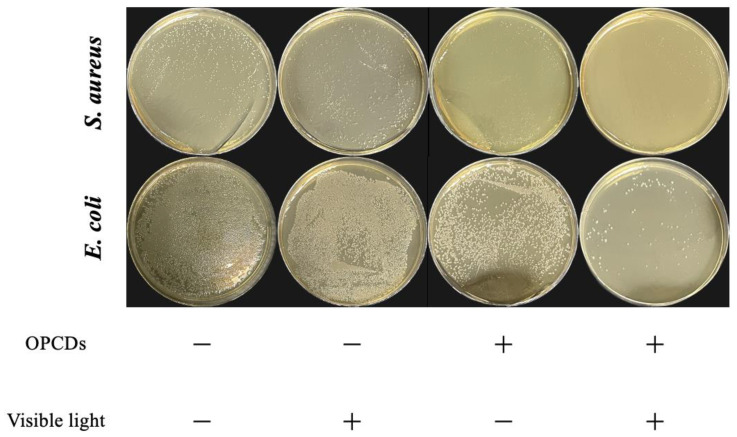
*S. aureus* and *E. coli* colonies treated with GACNPs with and without visible light (power density = 0.12 W/cm^2^).

To further investigate GACNPs, we evaluated the influence of photodynamic properties using the spread plate method and observation of the colony number. No evident changes were observed in the colony number of plates containing GACNPs with and without irradiation (Figure 5). *S. aureus* and *E. coli* were partially killed in the GACNP plates without irradiation. Meanwhile, GACNPs with irradiation killed all *S. aureus*, and *E. coli* survived partly owing to the photodynamic effect of irradiated GACNPs. The results confirm that GACNPs can provide phototherapy properties and enhance the antibacterial activity against both bacteria [57,75,76].

### 3.4. Detection of the Formation of Singlet Oxygen (^1^O_2_) of GACNPs

The generation of ROS was assessed by monitoring the reduction in absorbance intensity of the trapping agent, DPBF, as depicted in Figure S4. A significant decrease in DPBF absorbance intensity was observed, consistent with findings from a prior study [77]. DPBF reacts with singlet oxygen generated by GACNPs via a photochemical process known as the Diels–Alder reaction [78]. These results confirm the ability of GACNPs to produce ROS, which could ultimately lead to bacterial cell death.

### 3.5. Chemical Properties of the Hydrogels

**Figure 6:**
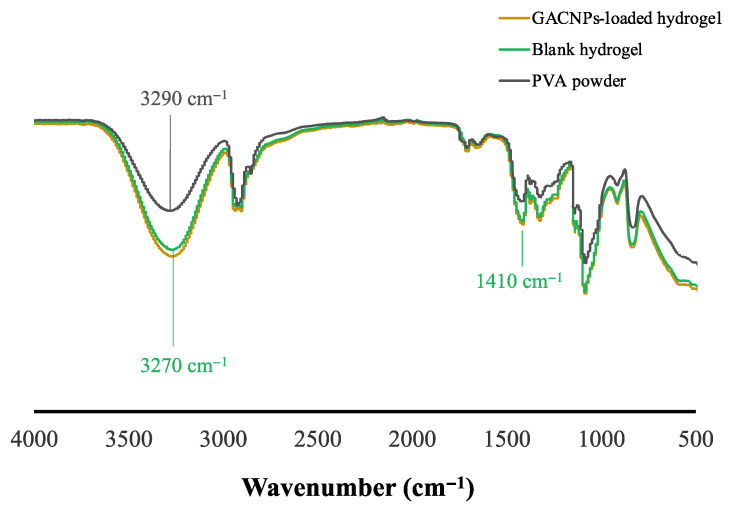
ATR-FTIR spectrum of PVA powder (black line), blank hydrogel (green line), and GACNP-loaded hydrogel (yellow line).

Figure 6 presents the ATR-FTIR spectra of the PVA powder, blank hydrogel, and GACNP-loaded hydrogel. The characteristic peaks of the blank hydrogel are as follows: The band at 3290 cm^−1^ is assigned to the stretching vibration of –OH, which presented an intermolecular force that induced the dipole–dipole attraction of the hydrogen bond between the H_2_O molecule and the hydroxyl group of PVA [79]. The intermolecular force can be used to confirm hydrogel fabrication. Because of an intermolecular force, the intensity around 3290 cm^−1^ is relatively increased, compared with PVA powder which is shown in Figure 6. Furthermore, a stretching vibration peak associated with the hydroxyl groups (–OH) in the crystalline regions of PVA is recorded at 3290 cm^−1^. This peak shifts slightly to 3270 cm^−1^ in the blank hydrogel. The observed hypsochromic (blue shift) in the –OH vibration indicates the disruption of PVA’s original crystalline structure and the formation of new hydrogen bonds between the hydroxyl group of PVA and the H_2_O molecule [80]. In addition, the peak at 1410 cm^−1^ that occurred between hydrogel interactions is assigned to the C–O group, which can be associated with the crystallite region [49,81,82]. Finally, the ATR-FTIR spectra of the GACNP-loaded hydrogel confirms that the loading of GACNPs into the hydrogel had no effect and retained the chemical properties of the blank hydrogel.

### 3.6. Mechanical Properties of the Hydrogels

Blank hydrogels of various gel thicknesses (1.7, 2.2, and 2.7 mm) were fabricated using a freeze–thaw technique. As shown in Table 3, the results reveal that 1.7 mm thick hydrogels showed the highest tensile strength and percent elongation; the increased gel thickness may result in inadequate cross-linking that causes the formation of thicker hydrogels [83,84]. By contrast, no significant differences were observed in the Young’s moduli under all thicknesses. Thus, the hydrogel with a thickness of 1.7 mm was selected for the loading of GACNPs. The amount of GACNPs varied at 0.5, 0.7, and 0.9 mg/g. The results show no significant differences in the tensile strength, elongation, and Young’s modulus of each GACNP/hydrogel patch. Therefore, the hydrogel patch loaded with 0.9 mg/g GACNPs was selected for further experiments because the largest amount can be loaded into the blank hydrogel without significantly changing the mechanical properties compared with the blank hydrogel. The hydrogel appearance of blank hydrogel and GACNP-loaded hydrogel patches is presented in Figure S5 of the Supplementary Materials file.

### 3.7. Water Content and Water Absorption

Table 4 shows the water content and water absorption of the hydrogel patches, including the blank and GACNP-loaded hydrogels. The results indicate that the water content of the hydrogel patches after loading GACNPs showed no considerable difference compared with that of the blank hydrogel before loading. By contrast, the water absorption of hydrogel patches after the loading of GACNPs was notably greater than that of blank hydrogels before loading because of the –OH functional groups on the surface of the GACNPs. The –OH group has the effect of weakening of the intermolecular force of the hydrogen bond, which caused the water molecules to permeate the GACNP-loaded hydrogel patches, as confirmed by the substantial increase in water absorption after loading [85].

### 3.8. Stability Study

The mechanical properties, water content, and water absorption of GACNP-loaded hydrogel patches were slightly changed in all storage conditions after 30 days, which is presented in Figure S7 of the Supplementary Materials file.

### 3.9. Antibacterial Activity of GACNP-Loaded Hydrogels

**Figure 7:**
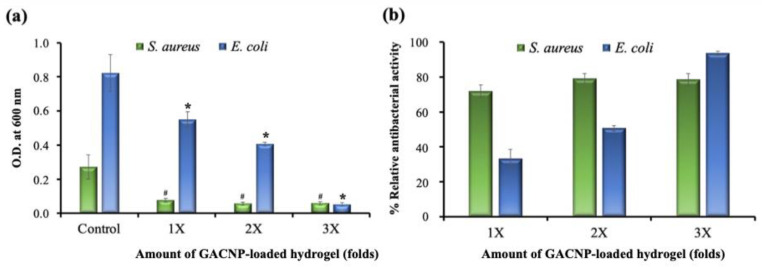
Antibacterial activity of different amounts of GACNP-loaded hydrogel patches on the growth of *S. aureus* and *E. coli.* (**a**) Inhibition effect after 24 h treatment and (**b**) % relative antibacterial activity. ^#^,* significant difference from the control of *S. aureus* and *E. coli* at *p* < 0.05.

The antibacterial activity of GACNP-loaded hydrogels was investigated quantitatively. GACNP-loaded hydrogels (0.08 g/piece) were added to the prepared bacterial suspensions in the amounts of 1, 2, and 3 pieces per well. After complete treatment for 24 h, the bacterial suspensions were collected to measure the turbidity at 600 nm. The results are shown in Figure 7a. The OD of the GACNP-loaded hydrogel patches decreased considerably after the treatment of bacterial suspensions. The more pieces used in the treatment, the higher the antibacterial activity exhibited compared with the control group. As shown in Figure 7b, the GACNP-loaded hydrogels inhibited *S. aureus* growth, with relative antibacterial activities of 71.85 ± 3.71%, 79.11 ± 2.98%, and 78.49 ± 3.54% for the amounts of 1X, 2X, and 3X, respectively. In addition, the GACNP-loaded hydrogels inhibited *E. coli* growth, with relative antibacterial activities of 33.09 ± 5.59%, 50.67 ± 1.46%, and 93.67 ± 1.20% for the amounts of 1X, 2X, and 3X, respectively. These results may be due to the fact that GACNP-loaded hydrogels can exhibit antibacterial properties against bacteria [86]. In addition, Gram-negative bacteria have an outer membrane preventing the penetration of foreign matter from the environment [87,88]. Therefore, the growth inhibition of *E. coli* required a higher amount of GACNPs than *S. aureus*.

**Figure 8:**
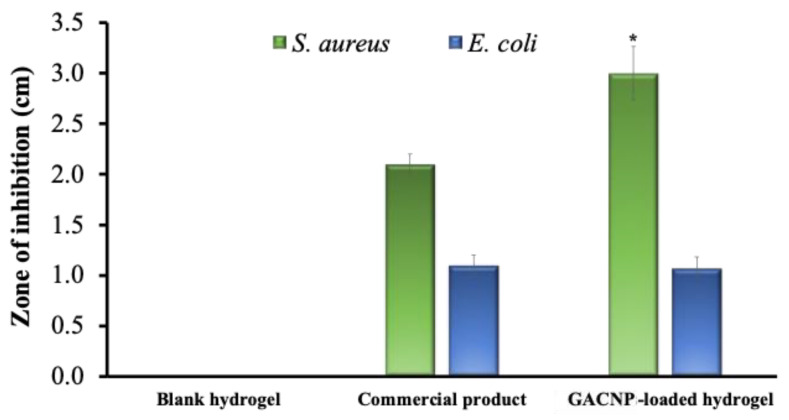
The inhibition zone of blank hydrogel, commercial product, and GACNP-loaded hydrogel against both *S. aureus* and *E. coli*. * Significant difference from the commercial product at *p* < 0.05.

For the qualitative analysis, the antibacterial activity of GACNP-loaded hydrogels, which were decorated at a weight of 0.08 g per piece, was estimated through disc diffusion assay. The blank hydrogel and commercial product (Ag hydrofiber^®^) were used as control groups. As shown in Figure 8, the results reveal that the blank hydrogel did not accelerate the zone of inhibition. GACNP-loaded hydrogels presented a zone of inhibition of 3.00 ± 0.26 cm against *S. aureus*, which was considerably greater than that of the commercial product (2.10 ± 0.10 cm zone of inhibition). By comparison, the antibacterial activity of GACNP-loaded hydrogels against *E. coli* was equal to that of the commercial product, with zones of inhibition of approximately 1.07 ± 0.12 and 1.10 ± 0.10 cm, respectively. The results reveal that compared with *E. coli*, *S. aureus* was more affected by GACNP-loaded hydrogels because Gram-positive bacteria can be damaged more easily by the negative charge of CNPs [89].

## 4. Conclusions

GACNPs were successfully fabricated through the microwave pyrolysis method. GACNPs can be loaded into hydrogel patches through a freeze–thaw procedure without substantially changing their properties. GACNPs can enhance the antibacterial activity against *S. aureus* and *E. coli*. Moreover, these NPs exhibit excellent photodynamic antibacterial activity against *S. aureus*. The GACNP-loaded hydrogel patches show superior antibacterial activity over a commercial product (Ag hydrofiber^®^). Furthermore, the water absorption of GACNP-loaded hydrogel patches, which is a good property for wound-dressing applications, is notably greater than that of blank hydrogels. These novel GACNPs can be potential antibacterial agents that may overcome multidrug-resistant problems in the future.

## Figures and Tables

**Table 1:** The effect of reaction time and temperature of GACNP synthesis by microwave pyrolysis method on particle size, PDI, and zeta potential of GACNPs.

Condition		Particle Size (nm)	PDI	Zeta Potential (mV)
Time (min)	Temperature (°C)			
10	200	286.3 ± 1.13	0.301 ± 0.052	−8.14 ± 0.30
15	200	238.9 ± 3.87	0.249 ± 0.011	−16.10 ± 1.27
20	200	96.91 ± 6.78	0.326 ± 0.013	−19.00 ± 1.91
20	175	139.5 ± 1.98	0.321 ± 0.114	−8.82 ± 1.67
20	220	128.6 ± 3.11	0.280 ± 0.049	−14.50 ± 3.32

The results are presented as mean ± SD.

**Table 2:** MIC and MBC of GACNPs against *S. aureus* and *E. coli* compared with GA solution.

Sample	*S. aureus*		*E. coli*	
	MIC (mg/mL)	MBC (mg/mL)	MIC (mg/mL)	MBC (mg/mL)
GACNPs	0.29	0.29	0.29	0.58
GA solution	1.24	1.24	1.24	1.24

**Table 3:** Mechanical properties of the blank hydrogel patches and GACNP-loaded hydrogel patches. The results are presented as mean ± SD (*n* = 3). (*) means statistically significant (*p* < 0.05).

Hydrogel Patches	GACNP-Loaded Hydrogel Patches (mg/g)	Thickness of Hydrogel (mm)	Young’s Modulus (Pa)	Tensile Strength (MPa)	Elongation (%)
	-	1.7	545.19 ± 0.29	0.35 ± 0.00 *	647.46 ± 8.90 *
Blank	-	2.2	563.32 ± 7.51	0.21 ± 0.00	378.17 ± 10.56
	-	2.7	563.66 ± 1.59	0.13 ± 0.01	218.57 ± 2.47
GACNPs	0.5	1.7	384.59 ± 9.44	0.19 ± 0.03	413.53 ± 15.57
GACNPs	0.7	1.7	355.37 ± 13.37	0.14 ± 0.00	395.71 ± 7.00
GACNPs	0.9	1.7	351.35 ± 10.19	0.15 ± 0.03	378.98 ± 11.88

**Table 4:** Water content and water absorption of the hydrogel patches. The data are expressed as mean ± standard deviation (*n* = 3). (*) means statistically significant (*p* < 0.05).

Hydrogel Patches	Water Content (%)	Water Absorption (%)
Blank hydrogels	82.17 ± 0.07	44.77 ± 6.47
GACNP-loaded hydrogels	74.70 ± 1.04	94.87 ± 2.90 *

## Data Availability

The data presented in this study are available in this article (and Supplementary Materials).

## Supplementary Materials

The following supporting information can be downloaded at: https://www.mdpi.com/article/10.3390/pharmaceutics16020254/s1, Figure S1: The photographic images of (a) gallic acid solution before synthesis and (b) GACNP dispersion; Figure S2: The photographic images of the histogram of the DLS of the nanoparticles; Figure S3: TGA thermograms of the GACNPs at a scan rate of 10 °C/min below 600 °C; Figure S4: The UV-vis absorbance of DBPF mixed with GACNPs at different light exposure times, 0 and 10 min; Figure S5: The photographic images of (a) blank hydrogel and (b) GACNP-loaded hydrogel patches; Figure S6: The photographic images of the inhibition zone of blank hydrogel, commercial product, and GACNP-loaded hydrogel against both (a) S. aureus and (b) *E. coli.* with visible light (power density = 0.12 W/cm^2^); Figure S7. The stability of GACNP-loaded hydrogel patches: (a) Water content (%), (b) Water absorption (%), (c) Young’s Modulus (Pa), (d) Tensile Strength (MPa), and (e) Elongation (%) of hydrogel patches kept at 5 °C, 25 °C, and 40 °C.
