# Supplementary material for: Photodynamic Antibacterial Therapy of Gallic Acid-Derived Carbon-Based Nanoparticles (GACNPs): Synthesis, Characterization, and Hydrogel Formulation
Source: Pharmaceutics. 2024 Feb 9;16(2):254. doi: 10.3390/pharmaceutics16020254 (PMC10891664; doi:10.3390/pharmaceutics16020254)
Supplement: Supplementary file 1 [file pharmaceutics-16-00254-s001.zip › pharmaceutics-2865445-supplementary.pdf]

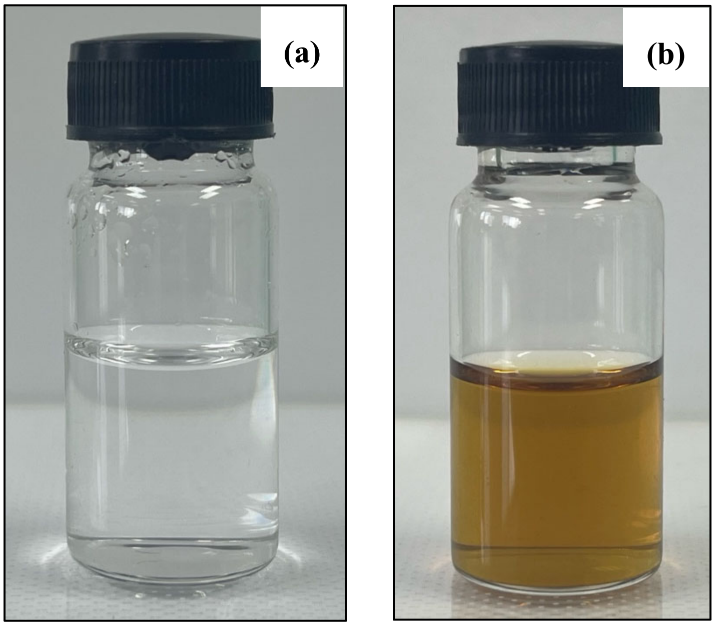

Figure S1. The photographic images of (a) gallic acid solution before synthesis and (b) GACNP dispersion.

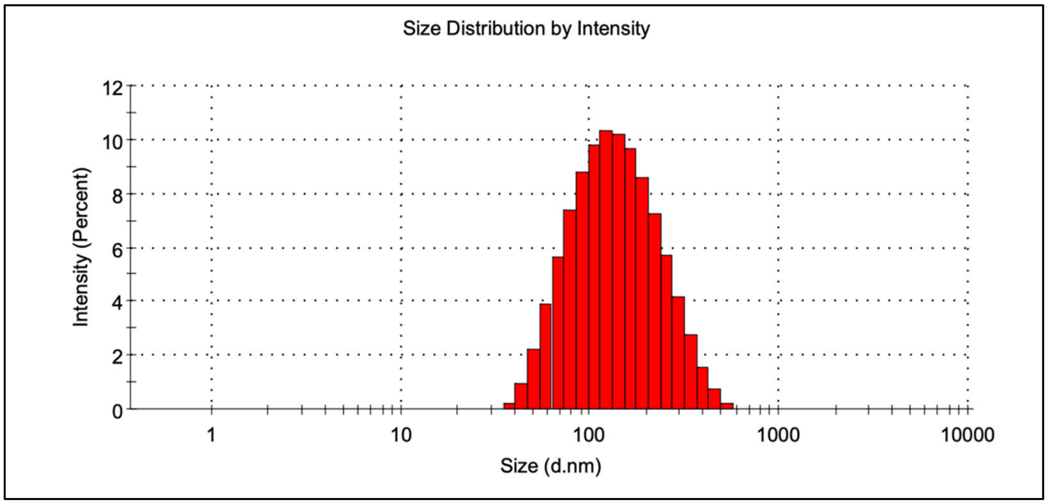

Figure S2. The photographic images of the histogram of the DLS of the nanoparticles.

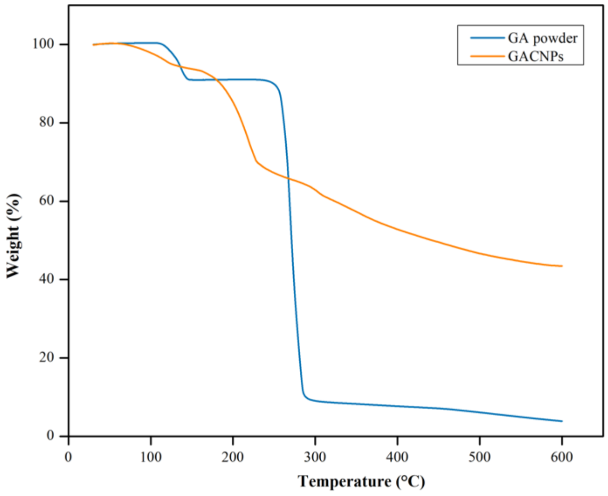

Figure S3. TGA thermograms of the GACNPs at a scan rate of 10 °C/min below 600 °C.

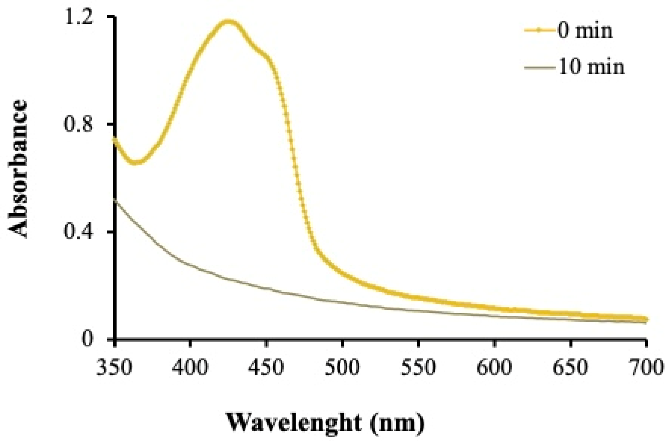

Figure S4. The UV-vis absorbance of DBPF mixed with GACNPs at different light exposure times, 0 and 10 min.

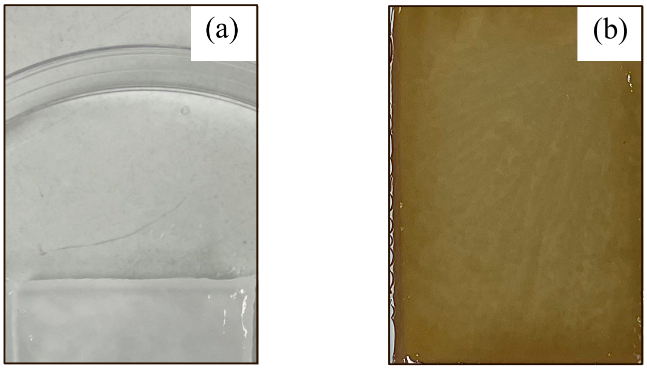

Figure S5. The photographic images of (a) blank hydrogel and (b) GACNP-loaded hydrogel patches.

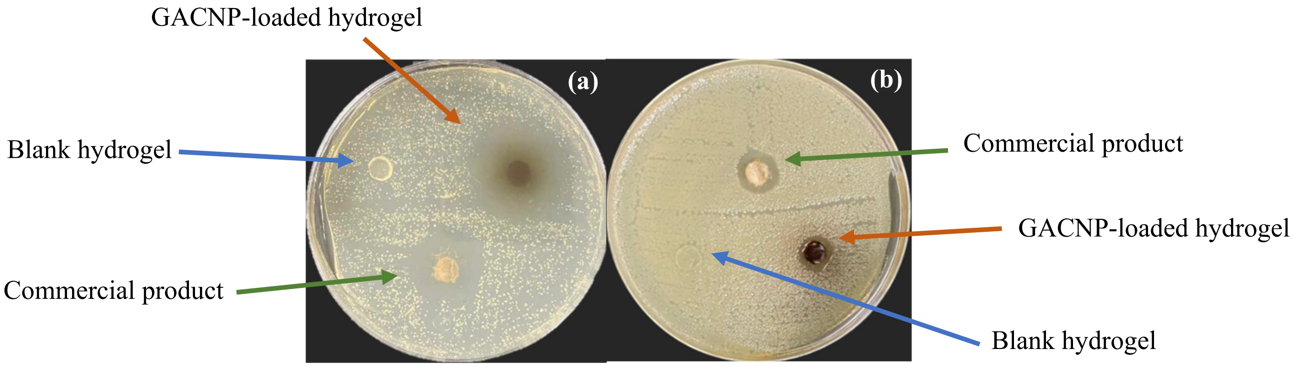

Figure S6. The photographic images of the inhibition zone of blank hydrogel, commercial product, and GACNP-loaded hydrogel against both (a) *S. aureus* and (b) *E. coli*. with visible light (power density = 0.12 W/cm^2).

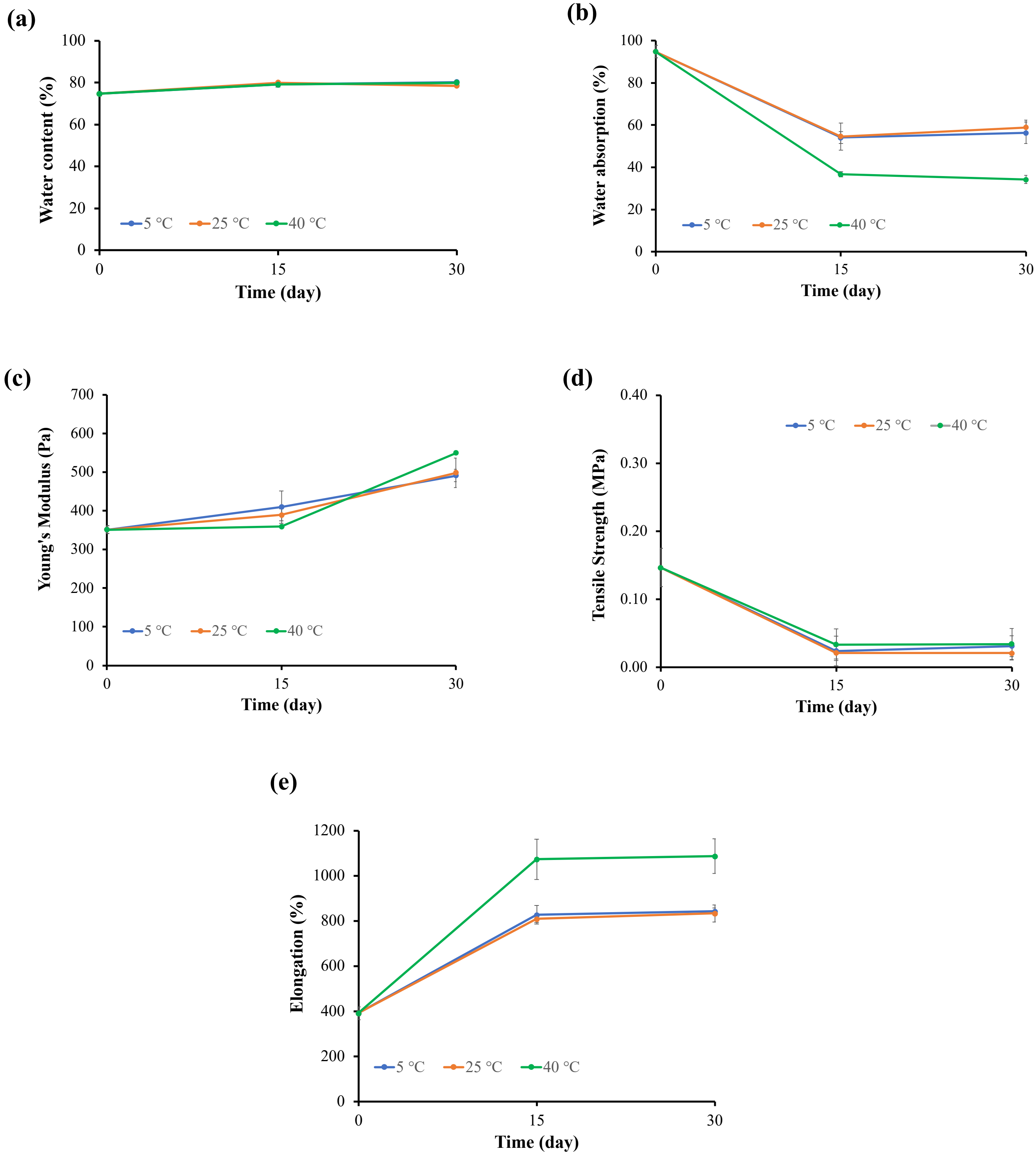

Figure S7. The stability of GACNP-loaded hydrogel patches: (a) Water content (%), (b) Water absorption (%), (c) Young's Modulus (Pa), (d) Tensile Strength (MPa), and (e) Elongation (%) of hydrogel patches kept at 5 °C, 25 °C, and 40 °C.
